# An Account of an Epidemic of Scarlatina, in Butler and Armstrong Counties, Pa.

**Published:** 1839-12-28

**Authors:** D. M. Borland

**Affiliations:** Freeport, Armstrong county, Pa.


### AN ACCOUNT OF AN EPIDEMIC OF SCARLATINA, *in Butler and Armstrong counties, Pa.*

BY D. M. BORLAND, M. D.

To the Editors of the Medical Examiner.

Gentlemen,—The perusal of several articles in your valuable journal on the subject of scarlatina, induces me to offer you the result of my experience in an epidemic of this disease, which has prevailed during the past summer in the southwestern part of Armstrong county, and in the southeastern part of Butler county, of this state.

Its first appearance was in the village of Freeport, where it presented an unusually malignant character. Of the malignant cases which were ushered in by vomiting and purging, two terminated fatally; one in twenty-two, and the other in fourteen hours after the first manifestation of the disease. In these cases there were great restlessness and prostration of strength, coma commencing a few hours after the attack, from which the system never reacted; very small and frequent pulse; the extremities became cold; the eyes turned up under the lids; the countenance sank; the lips became purple; the pupils dilated, and very sensible to light; with every manifestation of cerebral congestion.

One of these cases, which occurred in a child four months old, was protracted to the seventh day. There was extensive ulceration of the tonsils, extending to the posterior nares, giving rise to an acrid discharge, which excoriated the parts it came in contact with. The reaction was imperfect; the eruption appeared imperfectly on the fourth day, but soon disappeared; collapse now supervened; the heat of the surface began to sink; the pulse became very frequent and feeble; the tongue dark brown; sordes on the teeth; the animal powers entirely prostrated; to which succeeded convulsive twitchings of the extremities, and death.

The details of another fatal case will exhibit the different modifications of the disease, as it occurred in this epidemic.

A child, two years old, was attacked with the ordinary premonitory symptoms, followed by pain in the head; nausea and vomiting; pains in the loins and extremities, with general muscular prostration; an eruption, of a livid hue, made its appearance on the fourth day; the pulse, though in the commencement active, has now become small and feeble; delirium appeared with the eruption, and in a short time terminated in coma; the cheeks suffused with a livid flush; the eyes dull; dark-coloured sloughs appeared on the tonsils; the nose discharged an acrid fluid; the extremities became cold, and death closed the scene.

This epidemic commenced its ravages in the latter part of May, and continued until the 1st of December, under every variety, from the most mild to the most malignant form. No class or age appeared to be exempt from it; it attacked alike the child and the adult. I have prescribed for it in an infant of three weeks old; even persons who had formerly been the subjects of the disease suffered very much from sore throat.

*Treatment.*—In the congestive cases, my object was to equalize the circulation, and to arrest the violent vomiting and purging; for this purpose, mustard was applied to the stomach and extremities, together with rubifacients, composed of tinct. capsic. and aqua ammonia. As soon as reaction was established, I commenced with small doses of calomel, repeated every two hours until it operated on the bowels. Subsequently I gave cold-pressed castor oil, to keep up a regular but moderate evacuation of the bowels throughout the disease. As a gargle, I used pyroligneous acid and water, and chloride of soda and water, in the proportion of twelve parts of water to one of the other ingredients. With a view of lessening the swelling and soreness of the tonsils, I made use of equal parts of vinegar and turpentine, to be rubbed on until it produced an eruption, which it generally did in the course of a few hours, and with the happiest effect. After reaction was established, the skin became hot and dry, and the patient restless, I used, with the most gratifying results, cold vinegar and water, with which I ordered the patient to be sponged every half hour. This remedy appeared to act so promptly in soothing the patient, that frequently a calm and refreshing sleep was induced ere the sponging operation was completed, out of which the little sufferer awoke with all the symptoms mitigated. To aid the refrigerant effects of sponging, the patient was lightly covered, and cool air freely admitted into the chamber. During convalescence, which was generally rapid, I enjoined a light, but nourishing diet, and to guard carefully against the influence of cold and variable weather. The former part of the above treatment is adapted only to those cases in which there was a want of reaction. Such is the plan of treatment which I found most available in the present epidemic. In the commencement of the epidemic I resorted to emetics of ipecac., but they proved so unavailing that I subsequently abandoned them entirely. Indeed, sponging was the sheet-anchor of my hopes. As regards the dropsical affection which frequently supervenes, I have nothing to say, as it did not occur in a single case.

*Freeport, Armstrong county, Pa.*, Dec., 1839.

